# Description of the culture of childbirth and parenting classes in Cyprus: An ethnographic approach

**DOI:** 10.18332/ejm/186665

**Published:** 2024-06-03

**Authors:** Eleni Hadjigeorgiou, Maria Frangou, Yianna Koliandri, Maria-Dolores Christofi, Nicos Middleton

**Affiliations:** 1 Department of Nursing, School of Health Sciences, Cyprus University of Technology, Limassol, Cyprus

**Keywords:** perinatal, antenatal classes, parenthood, childbirth, pregnancy, education

## Abstract

**INTRODUCTION:**

Childbirth and parenting classes are very important because they potentially help couples to make the right decisions during pregnancy, childbirth, and the postpartum period, which has a direct effect on the health of the mother and neonate. However, in Cyprus, the culture of childbirth and parenting classes has not been previously explored.

**METHODS:**

An ethnographic study design was adopted, specifically non-participant observation was undertaken of 19 classes. Semi-structured telephone interviews were employed to collect data in addition to field notes and a reflective diary. Inductive content analysis was undertaken to analyze the data.

**RESULTS:**

Four main thematic categories emerged from data analysis: 1) Views and opinions about the course, 2) Important perinatal topics, 3) Usefulness and reasons for attending the classes, and 4) The journey of learning. The importance of antenatal classes has not been given sufficient attention in Cyprus.

**CONCLUSIONS:**

There is a clear need for a standardized curriculum within the current configuration of national maternity healthcare in Cyprus. Policymakers must implement a standardized curriculum, integrating diverse pedagogical methods to provide in-depth information for expectant parents and parents. While emphasizing the crucial role of midwives in perinatal education, this study also advocates for collaboration with other healthcare professionals emphasizing the imperative need for a comprehensive, standardized approach to perinatal education within the national healthcare system of Cyprus.

## INTRODUCTION

Pregnancy is normally a healthy condition during which a pregnant woman’s needs should be supported by appropriate evidence-based antenatal education, obstetric care and access to perinatal care services, to promote the health of their pregnancy and that of the fetus and neonate. Pregnant women, who are experiencing their first pregnancy, are those mostly in need of guidance and contact with midwives^1^. Antenatal classes, commonly known in Cyprus as childbirth and parenting classes, are a vital part of quality perinatal care, which prepares women for childbirth and helps couples take the right decisions during pregnancy, childbirth and postpartum^2^. The evidence highlights that these classes tend to have a positive effect on the health of childrearing women, and the couple, as they contribute to the reduction of anxiety in pregnant women and increase the participation rates of the partner in the delivery room^3^.

The antenatal classes provide participants with basic knowledge and skills in various aspects of maternal, fetal and neonatal health, aiming to reduce the risk of complications and enhance the positive experience of couples during childbirth^4^. International evidence suggests that in some countries antenatal classes do not meet the learning aims and educational needs of pregnant women^5^. Evidence from research in Australia demonstrates that the overall satisfaction of women who have attended antenatal classes was highly rated. Nevertheless, there were deficiencies in more than half of the variables studied, such as the lack of information on pregnancy issues and not being taken seriously by the midwife^6^.

It is common practice in developed countries to provide antenatal education as part of the perinatal care health scheme. In Cyprus, childbirth and parenting classes are offered by the national health system to parents-to-be who are planning childbirth at a public hospital. A few privately operated maternity units also offer childbirth and parenting classes to pregnant women and their partners, as part of the package of services offered to them during the perinatal period. Attendance is usually low in both cases.

Irrespective of international evidence revealing the evidence and strong support for antenatal education, evidence remains sparse from Cyprus and little is known about the experiences of antenatal education of women and their partners. This study was carried out to investigate how childbearing women and parents-to-be benefit from childbirth and parenting classes run in Cyprus, by exploring the culture of the participants during and after completion of classes, and employing ethnographic description methods. Furthermore, this study identifies the educational needs of women and parents-to-be and examines the contribution of the educator’s characteristics and learning environment in the satisfaction rates of the participants.

## METHODS

### Method and data collection

To provide in-depth insight and accurately describe the culture of childbirth and parenting classes, ethnography was selected as the most appropriate design method. An overt non-participant observation approach was employed at three separate entities in the town of Limassol: 1) the Limassol general hospital, 2) a private clinic, and 3) a non-governmental (private non-for-profit) organization ‘Baby Buddy Forward’. The researcher was present during the antenatal classes in an unobtrusive manner, observing and simply taking field notes, specifically recording the class, the interactions, the content and perception of participants feelings, and responses during the class. The participants were informed about the researcher’s role, but the researcher did not participate in the discussion and simply noted down field notes for the duration of the class. After each class the researcher used a reflective diary in order to reflect upon her experience in class. Data were collected using multiple methods commonly used in ethnography; using the researcher’s reflective diary, together with the field notes, the researcher recorded what happened at the place of the sessions, the attendance rate, the profile of the attendants, the activities and interactions of the attendants and the pregnant women’s feelings. Furthermore, telephone interviews with the pregnant women who participated in classes were conducted after the completion of these, using a semi-structured interview, which included open-ended questions. Twelve interviews were digitally recorded and transcribed verbatim. The interviews served to draw out detailed information about the participants’ experiences and perceptions, and open-ended questions allowed for women to express their experiences in whatever way was most meaningful to them. Data from the interviews were used to build upon the researchers’ observations, rich data were obtained.

### Data analysis

Analysis of the data was conducted using the inductive content analysis. The data transcription process involved verbatim transcription of interview transcripts and observational notes. Moreover, following Bennett et al.^7^ on qualitative data analysis, our study employed the inductive content analysis (ICA) approach. This method involves generating a comprehensive summary of individual texts within the dataset, and building analysis inductively through a thorough examination of the text rather than a predefined set of content items.

The analysis process commenced with coding, where sections of text within each transcript or document were systematically labeled. This coding methodology facilitated the identification and organization of similar text segments both within individual documents and across the dataset. Two researchers (MF, EH) conducted the coding process in an inductive manner, without imposing any pre-existing coding framework on the data. Initially, a multitude of codes emerged organically from the data reflecting various ideas expressed in the transcripts. This coding process was iterative and flexible, allowing codes to emerge freely from the dataset without predetermined constraints. The researchers then grouped these codes into categories and overarching themes through an iterative process of analysis. The development of themes evolved from the interconnections and relationships observed among these coded segments, ultimately culminating in a comprehensive thematic analysis of the dataset.

### Ethics

Ethics approval was obtained from the Cyprus National Bioethics Committee (ΕΕΒΚ ΕΠ 2018.01.124), the research committee of the Cyprus Ministry of Health and from the directors of the maternity departments of the public and private sectors involved in the study. All the participants were fully informed via email about the aim and purpose of the study, as well as the method of data collection. A written informed consent was obtained from the pregnant women participating in the study prior to interviewing them and were allocated a code in order to keep their identities confidential according to GDPR guidelines. They were also informed of their right to withdraw from the study at any time without any consequences.

## RESULTS

During the weekly childbirth and parenting classes, the demographic consisted of Greek-Cypriot pregnant women and their partners. Additionally, there were three Greek women, one Greek-American woman, and an English/ Scandinavian woman participating in the classes. The majority of the pregnant women were accompanied by their husbands or partners. A significant observation was the participants’ high education level, primarily at the tertiary level. Most of the participants seemed shy and were not demanding in class, while they only had very few enquiries or demands for clarifications. The majority of the participants seemed to prefer and liked to engage in general group discussions.

The qualitative data analysis of the documents resulted in four main themes: 1) Views and opinions about the classes, 2) Important perinatal topics, 3) Usefulness and reasons for attending the classes, and 4) The journey of learning. There were 14 sub-themes emerging from data analysis and these were grouped under the four main themes (Table 1).

**Table 1:** Themes and sub-themes

*Views and opinions about the classes*	*Important perinatal topics*	*Usefulness and reasons for attending the classes*	*The journey of learning*
Course organization	Pregnancy	Satisfaction level of learning needs	Sources of information
Educator characteristics	Birth	Socialization between participants	Finding reliable sources of information
Teaching method	Newborn care	Mutual support between couples	
Environment	The postpartum period		
	Childbirth rights		

### Views and opinions about the classes

The overall perceptions of pregnant women of the classes were mainly related to the way the course was organized, i.e. the class venue, the information given about the classes, the time of the day for the attendance of the classes, the duration of the classes, the characteristics and profile of the educator, and the teaching method used by the educator (lectures, hands-on workshops, demonstrations).

*Course organization*

The participating women seemed happy and appreciative of the small class size, which gave them the opportunity to socialize with other expectant parents and share their experiences. However, the majority expressed dissatisfaction with the venue, expressing the lack of adequate space and not being user-friendly. Additionally, many participants found the room decoration and overall environment irrelevant to the intended scope and purpose of the classes:

*‘It would be useful to prepare a room exclusively for this purpose … To include [pilates] balls, to include cots … and decorate that accordingly.’*

The duration of the classes was generally well-received by the participants. One participant mentioned:

*‘If it lasted any longer… I would not have been able to concentrate … just like it was timed … you were able to concentrate for two hours … you [the mind] did not wander about.’*

Additionally, most participants expressed satisfaction with the content of the classes, with one remarking:

*‘… the content was very good … because it covered preconception issues, nutrition during pregnancy, and more.’*

*Educator characteristics*

The educators played a crucial role in the organization of the classes. The participants described the educators as very approachable, supportive and helpful with regard to the various concerns of the women and the expectant parents. These characteristics created a climate of trust between the participants and the educator. Furthermore, the educators used motivational questions addressed to the class participants in order to encourage group discussions:

*‘The educators were very helpful. We could ask any kind of question … they were close to us, we did not feel uncomfortable … They were very approachable.’*

One of the venues attended by the researcher, employed educators who were specialized in the specific topics that they taught as healthcare professionals. It was apparent that the participants were able to trust them as a result of that:

*‘… due to their specialized knowledge … they provided us with scientifically based answers.’*

*Teaching method*

Predominantly, most of the educators, who were mainly midwives, used power-point presentations to deliver the content of the classes. In contrast, some educators used more inventive approaches incorporating various props, such as baby dolls and diapers, in order to assist them in the demonstration of certain skills. Additionally, some educators used multimedia resources such as photographic and video material of other couples’ experiences of childbirth. Some educators gave out written information such as leaflets on various topics, e.g. on the care of the infant, perinatal nutrition, breastfeeding etc.

*Environment*

The participants expressed a sense of comfort and intimacy in the atmosphere during classes:

*‘I’m comfortable, I mean all of us … with the educator and with the rest of the people, a pleasant atmosphere was created.’*

However, one pregnant woman said that the room was too crowded for her to feel comfortable asking questions:

*‘When there are too many [participants], I don’t express myself so easily. This is one of the reasons why I may have to stay in class after the lesson has finished to ask questions. So that others [participants] are not present.’*

### Important perinatal topics

*Pregnancy*

Questions and concerns were related to issues such as nutrition, gestational diabetes, exercise, and body care. A pregnant woman stated that she would have preferred to be informed about these issues at the start of her pregnancy, and not when she had reached the third trimester. The educators informed the women about certain foods and exercise that are prohibited during pregnancy and talked about exercise that is advisable during pregnancy, e.g. perineal exercises.

*Birth*

A burning question for pregnant women and their partners was childbirth and everything related to it. That is, the different types of birthing methods, the treatment of labor pain, the psychological empowerment of the women during childbirth, and the storage of umbilical cord blood. There was an apparent need for detailed information about the different modes of childbirth:

*‘Personally, I would like to know some specific information, let’s say … in other words, about the process of induced labor, how it is done … the pros and cons of a cesarean section.’*

Another popular topic was breathing techniques during childbirth, for the management of labor pain and anxiety. The women were very keen on practicing these in each lesson:

*‘… they [breathing techniques] helped me a lot … on the day that I went into labor, when I was very anxious …’*

*Newborn care*

Class content regarding the care of the newborn was mainly focused on feeding the newborn, i.e. artificial feeding, breastfeeding, and baby food. Vaccinations and psychosomatic development were also discussed in class. The couples were also keen in receiving training in first-aid skills for infants. First aid was not taught in the standard syllabus in any of the childbirth and parenting classes. First aid training was provided as a separate course, upon request, and was charged separately. The participants strongly expressed their willingness to attend first-aid classes, as their main worry was the possibility of their baby choking at some point.

A lot of the women’s queries about this topic were related to breastfeeding:

*‘Is the infant’s weight correlated with the amount of [breast] milk she/he will drink?’*

*‘… breastfeeding … I consider it very important, because … it is my first child and I don’t know how to handle breastfeeding … we were given a few details [in class] … that helped me a lot.’*

However, other women expressed the need for specific information about the practicalities and issues that may arise during breastfeeding:

*‘… a mother… told me that her breast milk stopped, because she didn’t massage her breasts and then she [the educator] advised us to apply compresses, massage the breasts, etc.’*

At one of the venues there was an interesting group discussion about whether other types of milk would be beneficial for the baby, such as goat or donkey milk.

Other topics discussed frequently in class were the baby’s bathing procedure, nappy changing, genital sanitation requirements for female and male infants.

There were questions about the bathing procedure and the optimum water temperature, the optimum room temperature that the baby should sleep in, questions about how heavily a baby should be dressed or covered, and how to massage a baby:

*‘… especially the lessons with the midwife on how to bathe a baby, were very useful.’*

The participants expressed their wish for more practical lessons on baby care, that would include practicing baby care using baby dolls, plastic bathtubs, nappies etc.

Information about vaccination protocols for infants was provided only at one of the venues attended by the researcher. The participants had many queries on this particular topic such as:

*‘What kind of vaccinations are not provided by the general health system?’*

*‘What are the mandatory vaccinations that the child should receive?’*

*‘Are booster doses necessary?’*

*The postpartum period*

The topics discussed related to the postpartum period of the woman included the puerperium, the resting needs of the mother, the mother’s psychology and the support mothers should receive from their husband/partner during puerperium. Sexual life after childbirth was also discussed. There was also a group discussion about contraception during breastfeeding, which was a topic that the couples seemed to be quite concerned about. There were several questions that were related to the mother’s nutrition needs during puerperium and the breastfeeding period. There was an emphasis on the fact that certain foods, such as legumes and vegetables, that cause bloating and gas, should be added gradually in the mother’s diet, in order to observe how these affect the infant during the breastfeeding period:

*‘My mother used to say that when [she] breastfed me and my brothers, [she] did not get pregnant. When she stopped, she became pregnant in one month.’*

*‘I know that breast feeding is not a contraceptive method. I know for sure because I read a book about that.’*

Also, a pregnant woman wondered:

*‘Does drinking beer help increase breast milk?’*

Following the previous question, a group discussion was triggered about alcohol, smoking and breastfeeding, which showed the participants’ varying beliefs and knowledge. The educator emphasized the detrimental impact of smoking and passive smoking on the baby’s health:

*‘I know that smoking is harmful but I did not know that passive smoking is harmful too. Now I know and I never allow anyone to smoke near me or my children.’*

This instance underscored the significance of education in transforming awareness and behavior, particularly regarding the impact of passive smoking on maternal and infant health.

*Childbirth rights*

The rights of pregnant women significantly impact on their overall quality of life that influences them throughout pregnancy and into the postpartum period. During the discussion, several pregnant women voiced their concerns regarding their rights during labor and childbirth. They emphasized the importance of having the autonomy to make decisions about their bodies and the well-being of their babies. One expectant mother expressed her distress, stating:

*‘I heard that some obstetricians did only cesarean section, some obstetricians or midwives did unnecessary intervention and sometimes failed to provide care which is needed to avoid preventable suffering, such as pain relief.’*

### Usefulness and reasons for attending the classes

*Satisfaction level of learning needs*

The women stated that they would recommend these classes to other parents-to-be and other pregnant women in the family and to friends who are expecting:

*‘Yes, of course I would recommend it [the course] to a friend of mine and the duration of the lesson was adequate … two hours and there was time to ask questions etc. … it was very good. No, I would not have to say anything negative about it.’*

*Socialization between participants*

Socializing and interacting with other women and couples who were going through the same ‘journey’ in life was as important as leaning the theory and practice of being a parent; this was quite evident when observing the behavior of the participants. The participants often empathized and interacted with each other, discussing issues of mutual interest, i.e. pregnancy, childbirth, baby care, vaccinations, breastfeeding and several other related topics; sharing their knowledge, emotions and experience. The women’s partners often laughed at each other’s jokes:

*‘To be honest with you, it is nice to know people who are going through the same phase as you, you feel … that you are not alone; that there are others who are also anxious about it … or who are preparing for this.’*

*Mutual support between couples*

The supporting role of the women’s partners throughout this process was very empowering and positive for the pregnant women. It was apparent that there was a mutual benefit for both the women and the men participating in the classes, as the men gained an understanding of a woman’s needs during pregnancy. During the class, the midwife would ask everyone to stand up, and would direct the husbands or the partners of the women to hold hands with the pregnant woman in order to practice patterned breathing. This was essential for the couple’s bonding during the prenatal period. The researcher noticed that most of the women’s partners were absent, when the educator planned to talk about breastfeeding. Considering the cultural context of the participants, the researcher hypothesized that this behavior might indicate the partners’ perception that breastfeeding issues primarily concerned the expectant mother and were not of their concern.

Moreover, during the lessons the researcher noticed the couples develop friendships between them. For example, they would talk like friends, they would worry if a pregnant woman did not attend the lessons. If one of the pregnant women felt anxious the other woman would ask them how they felt and how they could help them. One pregnant woman mentioned:

*‘One advantage for this lesson is that we come together and we can support each other.’*

### The journey of learning

*Sources of information*

The information and knowledge during the classes generally received positive feedback from participants, indicating a satisfactory level of understanding. However, participants also acknowledged the value of additional sources of information. A pregnant woman highlighted the practicality of technological applications, specifically mentioning the forthcoming launch of the ‘Baby Buddy Forward’ application by the Cyprus University of Technology:

*‘About what you said … the launch of an application “Baby Buddy Forward” that will be published by the Cyprus University of Technology … that would be very good, if it is promoted well enough in Cyprus … it would be very, very good. I have two [similar] applications … It counts my pregnancy weeks and informs me every week.’*

Interestingly, many participants identified online resources and relevant books on pregnancy and childcare as their primary sources of information. In each lesson, participants raised questions stemming from their readings, demonstrating a proactive engagement with external materials.

Interestingly, many women mentioned that the main source of information for them were online resources and relevant books on pregnancy and childcare. In each class, a lot of the participants raised questions in terms of information they received from the internet or in a book:

*‘I also watched various videos on bathing a baby, by midwives on YouTube. And everyone held the baby in a different manner, so I chose the one that was easiest for me [in practice].’*

*Finding reliable sources of information*

While participants acknowledged the variety of information available online, a prevailing concern was the uncertainty surrounding the reliability of these sources. Many pregnant women expressed hesitancy, questioning the credibility of information obtained from various online platforms.

They emphasized the importance of consulting healthcare professionals to verify and validate the information they came across. A participant highlighted this sentiment, stating:

*‘No matter how much you search … someone [a professional] who knows is, of course, more useful … because everybody says different things, so you never know what the right thing will be to do ultimately.’*

## DISCUSSION

The aim of this study was to explore the culture of childbirth and parenting classes in Cyprus through an ethnographic design. The findings reveal that the role of the midwife is pivotal in supporting expectant couples during these classes. Through an ethnographic approach, this study delved into a previously unexplored aspect of the childbirth and parenting landscape in Cyprus.

According to the researcher’s notes, reflective diary and interviews of the participating women, the impression is that, although the participants found the antenatal classes offered in Cyprus useful and valuable, their expectations remained unfulfilled.

The environment was an important parameter for the comfort and learning of the participants. Some of the pregnant women reported that the classroom was not ‘learner friendly’ because its arrangement was not suitable for such lessons, i.e. the chairs were not comfortable and appropriate for pregnant women and occasionally there were ‘too many’ participants in the room. Bondas^8^ conducted a study to explore women’s experiences of antenatal care, and reported that an aesthetic and comfortable environment (e.g. pictures and furniture in the room) makes pregnant women feel content.

The presence of other pregnant women in class offered women an opportunity for mutual support. It also seemed to empower women to face the expected risks and helped them receive comprehensive support, which in turn guided their own perinatal experience. The importance of social support in a similar setting, has been validated in the CenteringPregnancy^®^ group model of prenatal care^9^. The findings of Nolan^5^ support women’s preference for a smallgroup learning setting, in which they may interact with each other comfortably as well as with the educator and link the learning material to their personal situations.

The structure and content of the lessons were, overall, of satisfactory level. The participants appreciated that the information given to them was focused on evidence-based practice. Trusting the validity of the information given to the participating women seemed to be emotionally comforting for them. The importance of education in perinatal care, derived from evidence-based practice and the latest developments in its practice, is also emphasized by WHO^10^.

The educator’s characteristics played a major role on the views and opinions of the women and the engagement of participants during the course. At the ‘Baby Buddy Forward’ venue, it was evident that since the educators were professionals in the field that they taught, there was a sense of trust felt by the participants, as well as a more highlighted appreciation for the educator. Pregnant women mentioned that the attitudes, behaviors, skills and characteristics of the healthcare professionals are very important for them^11^.

Teaching was mostly conducted by oral presentation and occasionally supplemented by visual aids like photos and videos. Also, some instructors distributed printed material like leaflets or booklets, focusing on specific topics such as nutrition in pregnancy, neonate vaccinations, etc. While written resources were convenient for accessing validated information, certain participants expressed a preference for more interactive teaching methods. They sought engaging approaches such as practical demonstrations utilizing props and equipment, group activities, exercises, and role-playing sessions centered on infant care aspects like bathing, navel care, diaper changing, and feeding methods including both bottle and breastfeeding.

A recurrent theme that emerged was nutrition, many participating women had questions around nutrition. Midwives have an important role for the general provision of health advice, prenatal education and promotion of healthy eating^11^. Nevertheless, in this study it was apparent that the midwives did not have enough knowledge and skills to provide adequate or personalized advice about nutrition. Midwives need support from nutrition experts in order to provide nutrition advice for the promotion of a healthy pregnancy and to meet the individual needs of a pregnant woman^12^. First aid for infants was not included in the content of the classes, but it was highly requested by the participants. According to a study by Bassam^13^, the majority of mothers seem to recognize that first aid is important for those who have young children under the age of five years and that they should acquire first-aid skills. Furthermore, women acknowledge the use of the internet for information; a study also conducted in Cyprus using focus groups to explore the informational support needs and expectations of women, including the use of the internet, found that women do use the internet but often lack the skills to evaluate the information and need the guidance of healthcare professionals, as was evident in our study. In addition, it was found that women do ‘self-navigate in an unsupportive system’ highlighting the lack of services in terms of antenatal education where many women reported not knowing that these classes do exist^14^.

In the context of maternal health, the role of midwives extends beyond mere clinical assistance – it encompasses health counseling and education within the family and community. This responsibility involves preparing expectant parents through antenatal education and addressing broader aspects of women’s health, reproductive health, and child care. The research findings revealed a significant gap in the knowledge and expertise of the midwives conducting the perinatal classes. Many aspects covered in these classes lacked comprehensive understanding from the midwives, as was highlighted by the participants during their interviews. The participants expressed concerns about encountering multiple instances where the midwives fell short in adequately addressing their queries or providing in-depth information on various perinatal topics. Similarly, previous studies reported that most midwives did not recall receiving formal education or training on health literacy. Consequently, midwives might lack the necessary knowledge and skills to conduct formal health literacy assessments with pregnant women^15^.

A recurrent and important theme for participating women was the choice of mode of birth – whether to have a cesarean section or vaginal birth or water birth, which was also a popular topic during group discussions. The high rates of cesarean sections in Cyprus has led to the medicalization of childbirth during the last few years. According to Hadjigeorgiou et al.^16^, evidence-based, non-biased advice from healthcare professionals is a key factor which affects women’s choice of mode of birth. In comparison to countries with similar healthcare systems, the role of the midwife as an advocate for normal birth is not recognized or given sufficient attention in Cyprus^17^. Despite the potential for midwives to advocate normal childbirth in Cyprus, the existing midwifery model does not receive full acknowledgment. Even within public hospitals, the role of midwives remains under-recognized, compounded by the ongoing presence of obstetricians during normal births which undermines midwives’ autonomy^17^. Aksoy et al.^18^ report that women in Turkey requested elective cesarean sections due to fear of labor pain. Furthermore, the results suggested that the number of women with fear of childbirth, the severity of their fear, and the prevalence of elective cesarean sections, may all be reduced if pregnant women were better informed and educated about painless childbirth alternatives. A study comparing two groups of pregnant women – an antenatal education group and a control group – suggested that antenatal education has the effect of reducing the pregnant woman’s fear of childbirth and increases childbirth-related maternal self-efficacy^19^.

Within antenatal education is the need for genuine human connection and supportive relationships. Women actively seek opportunities to establish meaningful connections not only with other mothers but also with midwives. However, these desires for connection and supportive relationships often clash with the institutional setup of institution-based antenatal education which tends to align more with a fragmented biomedical model of care^20^. Additionally, midwives serve as vital advocates, providing emotional support and advocating for women’s choices during childbirth while also actively engaging in and understanding of the theoretical foundation of childbirth education techniques. However, some midwives face challenges experiencing feelings of helplessness or a sense of disconnection from the birthing process. Nevertheless, instances where midwives overly managed births were perceived negatively, hindering effective childbirth education technique implementation and potentially conflicting with women’s birth plans or preferences^21^.

### Limitations

The present qualitative study has attempted to portray and describe the culture of childbirth and parenting classes in Cyprus. However, the authors note that this study was limited to a small number of participants, residing in a single city (Limassol). Larger studies that include participants from many cities of Cyprus should be considered in the future.

## CONCLUSIONS

The aim of the study was to identify the needs of pregnant women and parents-to-be for more in-depth information and practical examples on important topics. Pregnant women expressed the need for realistic information, the importance of these topics being taught by healthcare professionals specializing in the field has been identified and highlighted in this study. Furthermore, this study reveals that the role of the midwife in perinatal education is imperative but also suggests that other related healthcare professionals could serve as facilitators to the midwife’s role.

The importance of antenatal classes needs to be supported more widely by healthcare professionals and policy makers, as attendance is low, compared to the number of primigravidas per year. Considering the health benefits of these classes, for the mother and child, policy makers should be concerned by this lack of attendance and furthermore implement a nationwide standardized curriculum, which will be provided within the framework of the national health system in Cyprus by midwives and other related health care professionals in the field. Any actions undertaken should take into consideration a woman centered approach, but also at the same time implement a family-oriented approach and the needs and expectations of the father-to-be equally, such as those of the mother and the neonate. Introducing childbirth and parenting classes in Cyprus can play a crucial role in promoting the health and well-being of expectant parents and their newborns. By providing education, empowerment, and community support, these classes can help couples make informed decisions and navigate the journey of pregnancy, childbirth, and early parenthood with confidence and resilience.

## Data Availability

The data supporting this research are available from the authors on reasonable request.
